# Effect of intermediate/high versus low dose heparin on the thromboembolic and hemorrhagic risk of unvaccinated COVID-19 patients in the emergency department

**DOI:** 10.1186/s12873-022-00668-8

**Published:** 2022-06-14

**Authors:** Claudia Marchioni, Gaetano Esposito, Mario Calci, Bruno Bais, GianLuca Colussi

**Affiliations:** 1 Division of Internal Medicine and Emergency Medicine Residency Program, Department of Medicine, University of Udine, 33100 Udine, Italy; 2 Department of Emergency Medicine, ASUFC University Hospital of Udine, 33100 Udine, Italy; 3 Thrombosis Prevention Unit, 2nd Division of Internal Medicine, Department of Medicine, ASUFC University Hospital of Udine, 33100 Udine, Italy

**Keywords:** SARS-CoV-2, Coronavirus, Survival analysis, Mortality, Anticoagulants, Hemorrhage

## Abstract

**Background:**

The optimal prophylactic dose of heparin in patients with coronavirus-associated disease 2019 (COVID-19) in the emergency department (ED) is debated. This study aimed to analyze different thromboprophylaxis approaches in unvaccinated COVID-19 patients admitted to ED without initial venous thromboembolism.

**Methods:**

Retrospectively, the effect of intermediate/high versus low dose heparin treatment was evaluated from December 2020 to July 2021 in a tertiary Academic Hospital in northeast Italy. The primary outcome comprised arterial or venous thromboembolism or all-cause death within 30 days. Secondary outcomes comprised each single primary outcome component or major hemorrhagic event. Cox regression was used to determine predictors of the primary outcome and propensity score weights to balance the effect of heparin treatment on all outcomes.

**Results:**

Data of 144 consecutive patients (age 70 ± 13, 33% females) were included in the study. High-dose prophylactic heparin was used in 69%, intermediate in 15%, and low in 17% of patients. The primary outcome occurred in 48 patients. Independent predictors of the primary outcome were COVID-19 severity (hazards ratio (HR) 1.96, 95% confidence interval (CI) 1.05–3.65, *p* = 0.035) and D-dimer levels (HR each log ng/dl 1.38, 95% CI 1.04–1.84, *p* = 0.026). Intermediate/high dose heparin did not affect the risk of the primary outcome compared with the low dose (weighted HR 1.39, 95% CI 0.75–2.56, *p* = 0.292). Intermediate/high heparin increased the risk of major hemorrhagic events (weighted HR 5.92, 95% CI 1.09–32, *p* = 0.039).

**Conclusions:**

In unvaccinated COVID-19 patients admitted to ED, prophylaxis with heparin at the intermediate/high dose did not reduce primary outcome compared with the low dose but increased the risk of major hemorrhagic events.

## Background

From May 2020 to July 2021, three major waves of “severe acute respiratory syndrome coronavirus 2” (SARS-CoV-2) infection occurred in Italy, with 3.6 million confirmed coronavirus-associated disease 2019 (COVID-19) cases and about 128.000 deaths of patients who were mostly unvaccinated [1]. In this period, because of the huge influx of symptomatic COVID-19 patients, the emergency department (ED) had a central role in the initial management of severe to critical diseases [2].

Clinical presentation of COVID-19 ranged from mild influenza-like symptoms to severe respiratory failure associated with thromboembolic complications that needed immediate oxygen, respiratory support, and anticoagulant treatments [3]. During the initial waves of the pandemic, unvaccinated patients were at high risk of disease progression toward severe to critical respiratory failure, hypoxia, venous and arterial thromboembolic complications, and death [4]. Venous thromboembolism (VTE) [5] and arterial thrombotic events (ATE) [6–8] were frequently observed in hospitalized patients with severe COVID-19 and both the complications contributed to the high mortality rate of these patients [9].

Although pathophysiological reasons for this frequent association are unknown, severe COVID-19 patients are characterized by bed rest, hypoxia, intense systemic and tissue inflammation, and abnormalities of coagulation markers [10–12]. These factors are expected to induce endothelial dysfunction, platelet activation, and blood stasis that justify the increased risk of VTE, meaning deep vein thrombosis (DVT) and pulmonary embolism (PE) [13]. In addition, virus infection induces a cytokine storm that can elicit arterial inflammation, local endothelialitis, hemostasis activation, and arterial thrombosis [14]. Mid-size artery thrombosis was shown in post-mortem lungs [15], as well as in other arterial sites, including coronary, cerebral, and peripheral arteries [16]. Because of this elevated thromboembolic risk, the use of prophylactic anticoagulants, such as heparin, in hospitalized patients with severe COVID-19 appeared mandatory [17, 18].

Although anticoagulants were mandatory to prevent thromboembolism and mortality in COVID-19 patients with severe to critical disease, the optimal prophylactic dose of heparin in the symptomatic COVID-19 patient without an initial VTE or ATE diagnosis is still a matter of debate [19]. The rational benefit of increasing doses of anticoagulants to prevent thromboembolic events and mortality is counterbalanced by the increased risk of major hemorrhagic events [20]. This study aimed to assess the risk/benefit ratio of different heparin doses regarding the incidence/risk of VTE, ATE, major hemorrhagic events, and mortality in a retrospective cohort of unvaccinated COVID-19 patients treated in ED.

## Methods

The design of this study was retrospective longitudinal. The study included data from patients consecutively admitted to the ED of the Academic Hospital of Udine in northeast Italy from December 2020 to June 2021. Patients were followed for 30 days and the study ended on July 2021. In this period, confirmed cases of SARS-CoV-2 infection, in the area served by the Academic Hospital, were 61,100 [21] and the vaccine prophylaxis was not commenced yet. Data of unvaccinated patients admitted to ED of all sexes, 18 years or older, with a positive nasal-pharyngeal swab for SARS-CoV-2 infection and clinical signs of moderate-to-severe pneumonia, and who had started prophylactic heparin within 24 h from admission were selected. Patients with an unclear swab response, who were discharged or died within 24 h from admission, needed anticoagulants for treating underline conditions, those with a platelet count lower than 50.000 cell/mm^3^, were on dialysis, or pregnant women were excluded. Patients with platelets below 50.000 cells/mm^3^ were excluded because they were not treated with prophylactic heparin because of their elevated bleeding risk.

According to WHO guidelines, a moderate disease was considered the presence of fever, cough, dyspnea, or fast breathing without alteration of vital signs; a severe disease, the addition of one of respiratory rate > 30 breaths/min, severe respiratory distress, or arterial oxygen saturation < 90% at room air [22]. All patients were examined at admission with at least a chest imaging [plain radiograph, ultrasound, or computed tomography (CT) scan] to assess pulmonary complications. A lung CT scan with contrast enhancement and a compressive venous ultrasound were performed whenever DVT or PE was suspected. All patients were treated with the best medical knowledge according to WHO guidelines available during the study period [22]. Information about patients' age, sex, comorbidities, drug therapy, smoking history, new-onset acute coronary syndrome, myocardial infarction, transitory ischemic attack, ischemic stroke, VTE, acute peripheral ischemia, major hemorrhagic events, and 30-day all-cause death was collected from clinical records.

Heparin doses were empirically prescribed by the emergency physician according to previous experience with anticoagulants in acutely ill medical patients [23] and evidence emerged from other observational studies in hospitalized COVID-19 patients [24]. Prophylactic heparin administration was differentiated according to the first prescription in “low dose” if it was equivalent to or lower than 4000 international units (IU) of enoxaparin administered once a day, “high dose” if used to 100 IU per kg of body weight taken twice a day, or “intermediate dose” if the dosage was in the middle of the previous two. We prefer using “low dose” instead of “prophylactic dose” and “high dose” instead of “therapeutic dose” since, in all patients, the first prescription of heparin was because of thromboprophylaxis. A heparin dosage lower than 4000 IU was prescribed when the estimated glomerular filtration rate (eGFR) was lower than 30 ml/min/1.73m^2^ to reduce the bleeding risk. The intermediate prophylactic dose was prescribed preferentially in obese patients or those with elevated D-dimer levels according to the experience of other groups [25].

The primary outcome of the study was the composite of ATE, VTE, or all-cause death within 30 days. ATE comprised myocardial infarction, ischemic cerebrovascular event, and acute peripheral artery ischemia. VTE comprised DVT and PE. Secondary outcomes comprised every single component of the primary outcome or major hemorrhagic events. Only for this study purposes, major hemorrhagic events were defined as whatever documented bleeding that induced the emergency physician to stop heparin treatment, whereas heparin-associated thrombocytopenia was suspected by platelets drop below 150.000 cells/mm^3^ or of 50% or more compared with pre-heparin baseline value [26]. An intention-to-treat approach based on the first dose prescription was used to analyze heparin effects on outcomes.

At admission, the following laboratory variables were collected: hemoglobin, platelet count, plasma D-dimer, plasma creatinine, and the prothrombin time expressed as the international normalized ratio (INR). The renal function was estimated by eGFR calculated with the Chronic Kidney Disease Epidemiology Collaboration (CKD-EPI) equation. CKD was defined when patients had a history of eGFR lower than 60 ml/min/1.73m^2^. Molecular testing for SARS-CoV-2 infection on nasopharyngeal swab samples was performed by real-time reverse transcriptase-polymerase chain reaction (RT-PCR) analysis of the virus RNA according to WHO guidelines [27]. Plasma levels of the cross-linked fibrin degradation product D-dimer were measured by latex enhanced immune-turbidimetric assay on an automated coagulation analyzer (ACL TOP, Instrumentation Laboratory). D-dimer concentration was reported as fibrinogen-equivalent-units (FEU). The certified laboratory service of the Academic Hospital of Udine performed all biochemical analyzes and routine blood tests by standard methods.

This study was performed under the ethical standards of the Declaration of Helsinki (1964) and its subsequent amendments and all methods were carried out under relevant guidelines and regulations. The Institutional Review Board of the University of Udine approved this study (Protocol Number 28–2022). All patients signed a generic informed consent to use personal data for research at hospital admission unless critically ill or deceased. The IRB stated that no additional specific informed consent for the retrospective analysis of patients’ data was needed.

### Statistical methods

Continuous normally distributed variables were summarized as mean and standard deviation (SD), whereas continuous not normally distributed ones as the median and interquartile range (IQR). The normal distribution of variables was assessed by looking at histograms and confirmed by the Shapiro–Wilk test. The mean difference of normally distributed variables was tested by Student’s t-test, whereas the difference between not normally distributed variables was tested with the Wilcoxon-Mann–Whitney test. Countable variables were organized in contingency tables and summarized by absolute count and proportions. The difference between proportions was tested with the exact Fisher’s test. For statistical purposes, heparin used at intermediate and high doses was considered in the same group. The effect of heparin treatment on primary and secondary outcomes was presented with the Kaplan-Maier curves and the non-parametric log-rank test statistic. Predictors of the primary outcomes were tested by the Cox proportional hazards regression. The multivariate model for the Cox analysis comprised variables that predicted the primary outcome with a probability (*p*) lower than 0.100 in the univariate analysis and the variable of interest “use of heparin at different doses” whatever *p* it showed. In the multivariate model, covariates of similar meaning or that highly correlated to each other were not included together. Not normally distributed variables were log-transformed before including them in univariate and multivariate analysis. Results of the Cox regression were reported as hazards ratio (HR) with a 95% confidence interval (CI). The effect of heparin at intermediate/high-dose versus low-dose (treatment effect) on the primary and secondary outcomes was presented as HR by Cox proportional hazards regression with robust standard errors [28], weighted for pretreatment unbalanced covariates with propensity score matching [29]. The propensity score for the average treatment effect of heparin in matched patients was estimated by logistic regression. Unbalanced covariates were considered those baseline variables whose standardized mean difference (SMD), between patients treated with heparin at intermediate/high versus low dose, was equal to or higher than 0.050 as an absolute value [30]. The balancing process was effective when it reduced below 0.050 the SMD value of each pretreatment covariate. The ideal sample size was estimated according to the heparin effect on the primary outcome by a Cox proportional hazards model. By considering 5% the alpha error and estimating 40% the proportion of patients using the intermediate/high dose heparin, 0.50 the expected HR for the treatment effect, 68 was estimated to be the minimum number of events needed to get a study power of 80%. Hypothesizing that 30% is the cumulative incidence of the primary outcome, the ideal sample size, should have been 227. The statistical analysis was performed with the free R software (version 4.1.2, R Core Team, Vienna, Austria) [31] and the packages Survival (version 3.2–13) [32] and WeightIt (version 0.12.0) [33].

## Results

After applying exclusion criteria, 144 consecutive patients were included in the study. The primary outcome occurred in 48 patients with a cumulative incidence of 33% and a median time to event of 8 days (IQR 4–11). A summary of patients’ general clinical and laboratory characteristics, the cumulative incidence of outcomes, and drug therapy are reported in Table 1. Three of the eight patients with ATE had an acute myocardial infarction, three ischemic strokes, and two acute peripheral ischemic limbs. The median time to ATE was 5 days (IQR 4–10). All nine patients with VTE had PE and only two had a concomitant DVT. The median time to VTE was 10 days (IQR 6–12). One of the seven patients with a major hemorrhagic event had gastrointestinal bleeding, five had a retroperitoneal hematoma, and one had an upper arm hematoma. The median time to hemorrhagic event was 9 days (IQR 8–14). All patients in this study were treated with enoxaparin and no heparin-induced thrombocytopenia have been diagnosed. The first dose prescription of heparin was continued until the study ended in 89 of 99, 17 of 21, and 16 of 24 patients who were taking, respectively, low, intermediate, and high doses. Heparin was escalated to the high dose in six and two patients who were taking, respectively, low and intermediate doses because a VTE event occurred. Only in one patient was a VTE event occurred despite the high dose of heparin and in this patient heparin was substituted with an oral anticoagulant. Heparin was substituted with oral anticoagulant also in four, two, and two patients who were taking, respectively, low, intermediate, and high doses because an ATE event occurred. The first dose of heparin was suspended in two and five patients who were taking, respectively, low and high doses because of the occurrence of a major hemorrhagic event.

**Table 1:** Comparison of variables between patients without and with primary outcome occurrence

Variable	All patients	Without primary outcome	With primary outcome	*p*
Patients (n)	144	96	48	
General clinical characteristics				
Age (years)	70 ± 13	68 ± 13	75 ± 12	0.002
Female sex (n (%))	48 (33)	31 (32)	17 (35)	0.712
Past or active smoker (n (%))	13 (9.0)	11 (11)	2 (4.2)	0.220
Comorbidities (n (%)):				
• Obesity	46 (32)	31 (32)	15 (31)	0.999
• Hypertension	88 (61)	54 (56)	34 (71)	0.105
• Diabetes	33 (23)	17 (18)	16 (33)	0.057
• Neoplasia	7 (4.9)	6 (6.3)	1 (2.1)	0.425
• Past coronary artery disease	18 (13)	11 (11)	7 (15)	0.601
• Past cerebrovascular disease	9 (6.3)	5 (5.2)	4 (8.3)	0.482
• Chronic kidney disease	59 (41)	33 (34)	26 (54)	0.031
Severe COVID-19 (n (%))	78 (54)	45 (47)	33 (69)	0.014
Outcomes				
All-cause death (n (%))	35 (24)	0	35 (73)	< 0.001
ATE (n (%))	8 (5.6)	0	8 (17)	< 0.001
VTE (n (%))	9 (6.3)	0	9 (19)	< 0.001
Major hemorrhagic event (n (%))	7 (4.9)	4 (4.2)	3 (6.3)	0.686
General biochemical variables				
Hemoglobin (g/dl)	13.4 ± 1.8	13.6 ± 1.5	13.0 ± 2.1	0.126
Platelet count (n. × 10^3^/mm^3^)	210 ± 82	218 ± 84	194 ± 76	0.099
Plasma creatinine (mg/dl)	1.02 (0.81–1.39)	0.98 (0.81–1.20)	1.15 (0.88–1.62)	0.011
eGFR (ml/min/1.73m^2^)	66 ± 29	71 ± 27	55 ± 30	0.004
Plasma D-dimer (ng/dl FEU)	771 (545–1704)	692 (521–1302)	1240 (722–2613)	0.002
Prothrombin time (INR)	1.10 (1.56–1.19)	1.10 (1.045–1.21)	1.13 (1.06–1.18)	0.776
Drug therapy				
Heparin use (n (%))				
•Low dose	99 (69)	69 (72)	30 (63)	0.260
•Intermediate dose	21 (15)	13 (14)	8 (15)	0.812
•High dose	24 (17)	14 (15)	10 (21)	0.352
Antiplatelet drugs (n (%))	49 (34)	27 (29)	22 (42)	0.143
Statins (n (%))	34 (24)	25 (26)	9 (19)	0.408

*ATE*, arterial thrombotic event, *VTE* venous thromboembolism, *eGFR* estimated glomerular filtration rate by the Chronic Kidney Disease Epidemiology Collaboration (CKD-EPI) equation, *FEU* fibrinogen equivalent units, INR, international normalized ratio, *p*, probability

Patients with the primary outcome were older, had more frequently CKD and severe COVID-19, and had lower eGFR, but higher plasma D-dimer and creatinine levels than patients without (Table 1). Patients treated with heparin at the intermediate/high dose were more frequently obese and presented more hemorrhagic events than those treated with the low dose (Table 2). Positive predictors of the primary outcome in the univariate Cox regression were age, diabetes, CKD, severe COVID-19, and D-dimer levels, whereas the only negative predictor was eGFR (Table 3). In the multivariate model, all but one positive and negative predictor of the primary outcome with *p* lower than 0.100 were included. The variable CKD was excluded because of its high correlation with eGFR. Independent predictors of the primary outcome remained COVID-19 severity and plasma D-dimer levels (Table 3).

**Table 2:** Difference between variables of patients treated with intermediate/high versus low dose heparin

Variable	Low heparin dose	Intermediate/high heparin dose	*p*	SMD
Patients (n)	99	45	-	-
Age (years)	70 ± 13	71 ± 13	0.532	0.019
Female sex (n (%))	36 (36)	12 (27)	0.340	-0.219^a^
Past or active smoker (n (%))	10 (10)	3 (6.7)	0.755	-0.137^a^
Comorbidities (n (%)):				
• Obesity	24 (24)	22 (49)	0.006	0.493^a^
• Hypertension	56 (56)	32 (71)	0.140	0.321^a^
• Diabetes	23 (23)	10 (22)	0.999	-0.024
• Neoplasia	6 (6.0)	1 (2.2)	0.435	-0.260^a^
• Past coronary artery disease	13 (13)	5 (11)	0.999	-0.064
• Past cerebrovascular disease	6 (6.0)	3 (6.7)	0.999	0.024
• Chronic kidney disease	39 (39)	20 (44)	0.588	0.102^a^
Severe COVID-19 (n (%))	49 (49)	29 (64)	0.107	0.312^a^
Hemoglobin (g/dl)	13.3 ± 1.7	13.7 ± 1.8	0.140	0.266^a^
Platelet count (n. × 10^3^/mm^3^)	217 ± 90	194 ± 56	0.063	-0.410^a^
eGFR (ml/min/1.73m^2^)	67 ± 30	63 ± 27	0.486	-0.129^a^
Plasma D-dimer (ng/dl FEU)	733 (561–1367)	1075 (522–1789)	0.581	0.047
Prothrombin time (INR)	1.11 (1.06–1.19)	1.10 (1.05–1.22)	0.894	-0.083^a^
Antiplatelet drugs (n (%))	33 (33)	16 (36)	0.850	0.046
Statins (n (%))	24 (24)	10 (22)	0.836	-0.048
Outcomes				
Primary outcome (n (%))	30 (30)	18 (40)	0.260	-
All-cause death (n (%))	24 (24)	11 (24)	0.999	-
ATE (n (%))	4 (4.0)	4 (8.9)	0.257	-
VTE (n (%))	6 (6.0)	3 (6.7)	0.999	-
Major hemorrhagic event (n (%))	2 (2.0)	5 (11)	0.031	-

*ATE* arterial thrombotic event, *VTE* venous thromboembolism, *eGFR* estimated glomerular filtration rate by the Chronic Kidney Disease Epidemiology Collaboration (CKD-EPI) equation, *FEU* fibrinogen equivalent units, *INR* international normalized ratio; *p* probability, *SMD* standardized mean difference

^a^Unbalanced covariates with SMD higher than 0.050 absolute value. SMD for skewed variables was calculated on log-transformed values

**Table 3:** Univariate and multivariate Cox proportional hazards analysis of predictors of the primary outcome

	**Univariate analysis**		**Multivariate analysis**	
**Variable**	**HR (95% CI)**	***p***	**HR (95% CI)**	***p***
Age (each 10 years)	1.28 (1.01–1.61)	0.042	1.13 (0.89–1.44)	0.319
Female sex (yes/no)	1.15 (0.64–2.08)	0.642	-	-
Past or active smoker (yes/no)	0.39 (0.10–1.62)	0.195	-	-
Comorbidities (yes/no):				
• Obesity	0.99 (0.54–1.82)	0.971	-	-
• Hypertension	1.67 (0.90–3.12)	0.106	-	-
• Diabetes	1.92 (1.05–3.49)	0.034	1.30 (0.68–2.50)	0.430
• Neoplasia	0.34 (0.05–2.47)	0.287	-	-
• Past coronary artery disease	1.36 (0.61–3.02)	0.456	-	-
• Past cerebrovascular disease	1.22 (0.46–3.60)	0.623	-	-
• Chronic kidney disease	1.87 (1.06–3.30)	0.031	-	-
COVID-19				
• Moderate (Reference)	1		1	
• Severe	2.09 (1.13–3.85)	0.018	1.96 (1.05–3.65)	0.035
Hemoglobin (each 1 g/dl)	0.86 (0.73–1.02)	0.072	0.97 (0.80–1.18)	0.783
Platelet count (each 10^4^/mm^3^)	0.97 (0.93–1.01)	0.157	-	-
eGFR (each 10 ml/min/1.73m^2^)	0.87 (0.78–0.96)	0.005	0.94 (0.83–1.06)	0.308
D-dimer (each log ng/dl FEU)	1.55 (1.20–2.00)	< 0.001	1.38 (1.04–1.84)	0.026
Prothrombin time (each log INR)	5.29 (0.74–38)	0.097	5.72 (0.84–39)	0.075
Heparin use				
• Low dose (reference)	1		1	
• Intermediate/high dose	1.47 (0.82–2.63)	0.200	1.44 (0.79–2.62)	0.237
Antiplatelet drugs (yes/no)	1.44 (0.81–2.55)	0.216	-	-
Statins (yes/no)	0.74 (0.36–1.53)	0.420	-	-

*eGFR* estimated glomerular filtration rate by the Chronic Kidney Disease Epidemiology Collaboration (CKD-EPI) equation, *FEU* fibrinogen equivalent units, *INR* international normalized ratio, *HR* hazards ratio, *CI* confidence interval, *p* probability

**Fig. 1:**
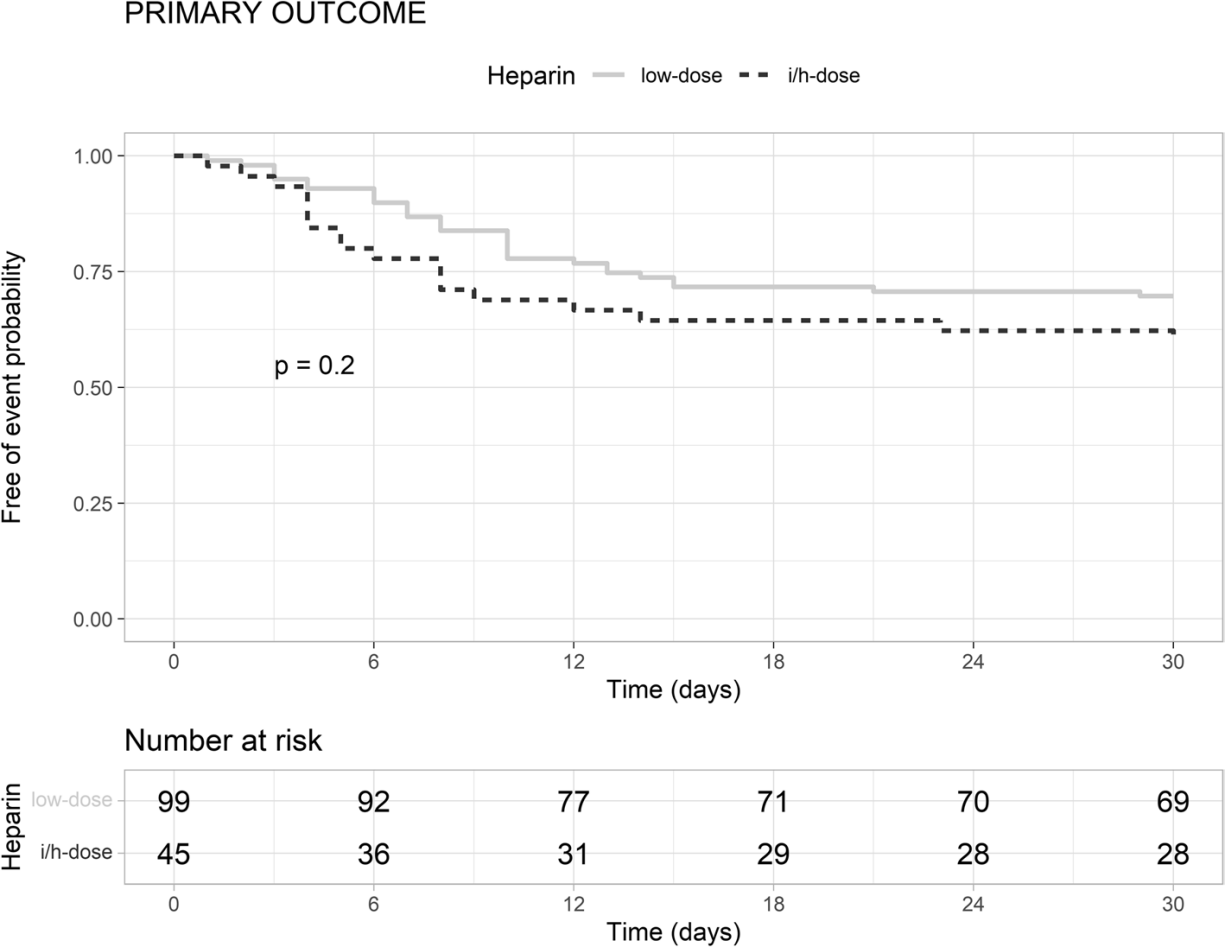
Kaplan-Meyer curves of the effect of heparin treatment on the primary outcome. In the graph is reported the probability (p) of the log-rank test. i/h, intermediate/high

**Fig. 2:**
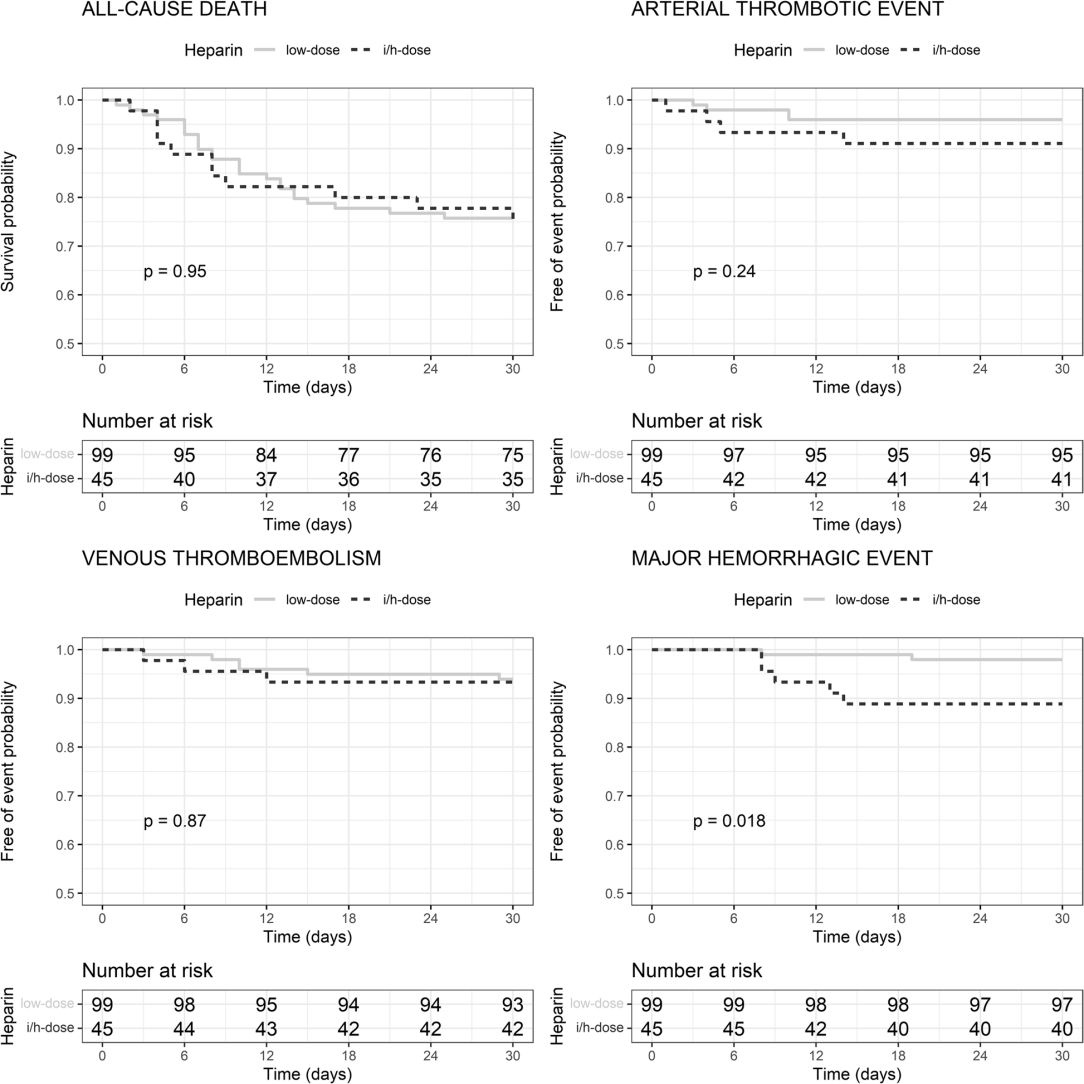
Kaplan-Meyer curves of the effect of heparin treatment on secondary outcomes. In the graph is reported the probability (p) of the log-rank test. i/h, intermediate/high

**Fig. 3:**
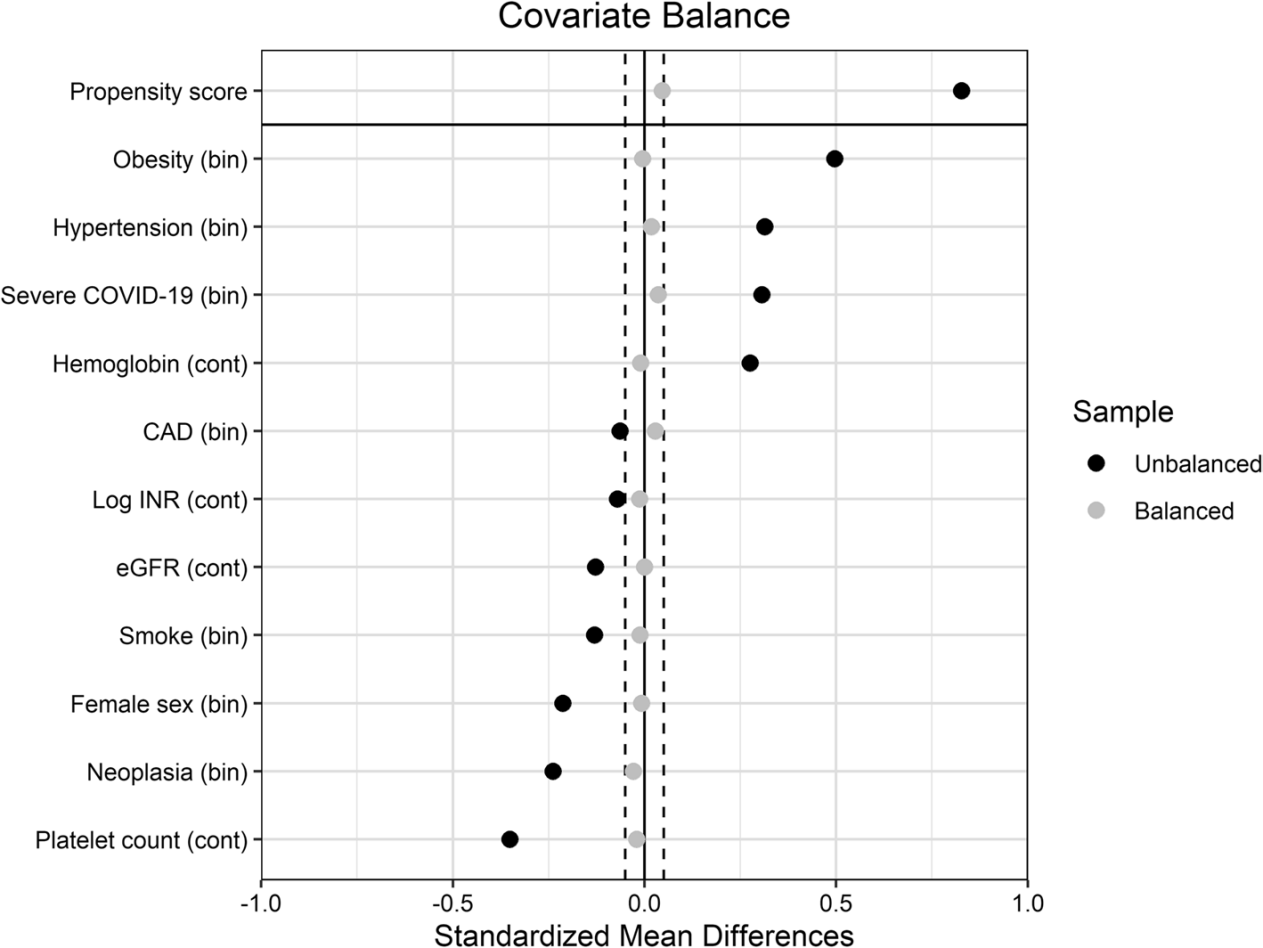
Effect of the propensity score weighting on unbalanced pretreatment covariates. Vertical dashed lines represent the standardized mean difference limit of ± 0.050. Bin, binary variable; cont, continuous variable; CAD, coronary artery disease; INR, international normalized ratio (prothrombin time); eGFR, estimated glomerular filtration rate by the Chronic Kidney Disease Epidemiology Collaboration (CKD-EPI) equation

Figures 1 and 2 present the Kaplan–Meier curves and the log-rank test statistic of the heparin effect on the primary and secondary outcomes. Using heparin at the intermediate/high dose compared with the low dose did not reduce the probability of the primary outcome (Fig. 1), whereas it increased that of major hemorrhagic events (Fig. 2). Unbalanced pretreatment covariates, were female sex, past or active smoker, obesity, hypertension, neoplasia, CKD, severe COVID-19, hemoglobin, platelets, eGFR, and INR (Table 2). Figure 3 presents the effect of the propensity score weighting on unbalanced covariates. After applying propensity score weighs on the Cox regression, the use of heparin at intermediate/high dose compared with the low dose did not reduce the risk of the primary outcome (weighted HR 1.38, 95% CI 0.75–2.56, *p* = 0.292), all-cause death (weighted HR 1.03, 95% CI 0.49–2.18, *p* = 0.929), ATE (weighted HR 1.78, 95% CI 0.38–8.3, *p* = 0.465), and VTE (weighted HR 0.94, 95% CI 0.23–3.89, *p* = 0.929), whereas it increased that of major hemorrhagic events (weighted HR 5.92, 95% CI 1.09–32, *p* = 0.039). The median time to hemorrhagic event in patients treated with heparin at intermediate/high dose was 9 days (IQR 8–13), and that of patients treated with the low dose was 14 days (IQR 11–16).

## Discussion

In this study, it was shown that heparin at the intermediate/high dose compared to the low dose did not reduce the risk of VTE, ATE, or all-cause death within 30 days. Conversely, the intermediate/high dose increased the risk of major hemorrhagic events.

This study reported the clinical experience of an ED in northeast Italy during the initial COVID-19 pandemic when almost all patients were unvaccinated and evidence on anticoagulation strategies was poor. Therefore, heparin prophylaxis was administered according to the general knowledge about the VTE risk in hospitalized and critical septic patients [34]. Nevertheless, the results confirmed what more recent prospective studies and randomized controlled trials were showing [35].

This study was conducted during the second and third waves of the SARS-CoV-2 pandemic in Italy when virus mutations became dominant [36]. In this period, the wild-type Wuhan virus, responsible for the Italian first wave in early 2020, was rapidly substituted by the United Kingdom (Alpha) variant (Phylogenetic Assignment of Named Global Outbreak lineages B.1.1.7) more infective [37], but less lethal [36]. Although patients in this study were not genotyped for SARS-CoV-2 variants, the Italian National Institute of Health periodically performed nationwide surveillance of virus variants according to random sampling from reference regional laboratories. The second bulletin of variants prevalence in Italy, including data between 28 December 2020 and 6 June 2021, estimated that the prevalence of the Alpha variant (lineage B.1.1.7) in northeast Italy, the area of patients’ provenience, was predominant (96.3%) [38].

In critical COVID-19 patients, usually admitted to the intensive care unit (ICU), high dose prophylactic heparin without a documented VTE is not recommended, whereas in hospitalized non-ICU patients it should be considered after having evaluated the bleeding risk [39]. The REMA-CAP et al. investigators' trial on ICU patients was prematurely stopped because prophylactic heparin at a therapeutic dose compared with the usual prophylaxis reduced survival at hospital discharge and the number of days free of cardiovascular and organ support and induced a 65% increment of major bleeding [40]. On the opposite, the ATTAC Investigators trial [41] showed the beneficial effect of the prophylactic higher doses of heparin compared with the low dose in COVID-19 associated with adverse outcomes and mortality in hospitalized non-ICU patients. In all these studies, higher doses of prophylactic heparin increased major bleedings or thrombocytopenia compared with the low dose, though the cumulative incidence of hemorrhagic events was low and not always statistically significant. Flumignan et al. summarize these results in a meta-analysis showing that higher doses of anticoagulants increase the relative risk of major bleeding by 78% (95% CI 13%-280%), and reduce that of PE by 54% (95% CI 30%-69%), without affecting all-cause mortality, risk of DVT, and need for additional respiratory support [35].

Unexpectedly, in the present study, higher doses of heparin did not reduce the risk of VTE events compared to the low dose. This observation is not uncommon since also in other studies VTE occurred despite thromboprophylaxis with elevated doses of heparin [42]. A potential mechanism that could explain this discrepancy is “heparin resistance”, reduced activity of the drug against the coagulation factor Xa [43]. Although this potential negative effect of COVID-19 on heparin efficacy is unexplained, this condition has been observed in about 75% of critical COVID-19 patients and it cannot be excluded that some patients in the present study might have presented heparin resistance during treatment.

The most important limitation of this study is that it did not reach the number of minimum events of the primary outcome to get power above 80%. However, looking at the survival curves, the use of heparin at the intermediate/high dose was far from being superior compared with the low dose to reduce the risk of the primary outcome, whereas the use of heparin increased the hemorrhagic risk with more solid evidence. Nevertheless, the findings of this study are in line with that of other groups and the most recent evidence. The second limitation of this study is the retrospective design. Because patients were not included with an a priori evaluation, selection bias introduced an elevated heterogeneity, as documented by the large standard deviation of some variables and by the large confidence intervals of estimates. To reduce this problem, multivariate analysis was adjusted by including relevant confounders with a *p* lower than 0.100 (not the conventional 0.050) and the heparin effect was weighted with a propensity score to be more conservative. The third limitation is the lack of a standard protocol for heparin use. Many patients started prophylactic heparin according to clinician self-assessment and, in some cases, heparin doses were changed during the follow-up because of the onset of new clinical needs. The effect of these changes on the study outcomes was not considered because it was not an object of this study.

## Conclusions

In this study, prophylactic heparin at a high/intermediate dose did not reduce the primary outcome compared with the low dose but increased the risk of major hemorrhagic events. With the important limitations exposed in the previous paragraph, this study does not support the use of intermediate/high dose heparin instead of the low dose for thromboprophylaxis in ED.

## Data Availability

The datasets generated and analyzed during the current study are available from the corresponding author on reasonable request and after authorization by the Administrative Department of the University of Udine.

## Abbreviations

SARS-CoV-2: Severe Acute Respiratory Syndrome Coronavirus 2
COVID-19: Coronavirus-associated Disease 2019
ED: Emergency Department
VTE: Venous Thromboembolism
ATE: Arterial Thrombotic Event
PE: Pulmonary Embolism
DVT: Deep Vein Thrombosis
CKD: Chronic Kidney Disease
eGFR: Estimated Glomerular Filtration Rate
FEU: Fibrinogen Equivalent Unit
INR: International Normalized Ratio
HR: Hazards Ratio
CI: Confidence Interval
ICU: Intensive Care Unit

## References

[1] Ritchie H, Mathieu E, Rodés-Guirao L, Appel C, Giattino C, Ortiz-Ospina E, et al. "Coronavirus Pandemic (COVID-19)". Published online at OurWorldInData.org. https://ourworldindata.org/coronavirus.

[2] Lopez Bernal J, Andrews N, Gower C, Robertson C, Stowe J, Tessier E (2021). Effectiveness of the Pfizer-BioNTech and Oxford-AstraZeneca vaccines on covid-19 related symptoms, hospital admissions, and mortality in older adults in England: test negative case-control study. BMJ.

[3] Khan M, Adil SF, Alkhathlan HZ, Tahir MN, Saif S, Khan M (2020). COVID-19: A Global Challenge with Old History. Epidemiology and Progress So Far Molecules.

[4] Grima AA, Murison KR, Simmons AE, Tuite AR, Fisman DN. Relative Virulence of SARS-CoV-2 Among Vaccinated and Unvaccinated Individuals Hospitalized with SARS-CoV-2. Clin Infect Dis. 2022;ciac412. 10.1093/cid/ciac412. 10.1093/cid/ciac412 PMC9278128 35616115

[5] Suh YJ, Hong H, Ohana M, Bompard F, Revel M-P, Valle C (2021). Pulmonary Embolism and Deep Vein Thrombosis in COVID-19: A Systematic Review and Meta-Analysis. Radiol.

[6] Vogrig A, Gigli GL, Bnà C, Morassi M (2021). Stroke in patients with COVID-19: Clinical and neuroimaging characteristics. Neurosci Lett.

[7] Tomerak S, Khan S, Almasri M, Hussein R, Abdelati A, Aly A (2021). Systemic inflammation in COVID-19 patients may induce various types of venous and arterial thrombosis: A systematic review. Scand J Immunol.

[8] Harrison SL, Buckley BJR, Rivera-Caravaca JM, Zhang J, Lip GYH (2021). Cardiovascular risk factors, cardiovascular disease, and COVID-19: an umbrella review of systematic reviews. Eur Heart J Qual Care Clin Outcomes.

[9] Poor HD (2021). Pulmonary Thrombosis and Thromboembolism in COVID-19. Chest.

[10] Panigada M, Bottino N, Tagliabue P, Grasselli G, Novembrino C, Chantarangkul V (2020). Hypercoagulability of COVID-19 patients in intensive care unit: A report of thromboelastography findings and other parameters of hemostasis. J Thromb Haemost JTH.

[11] Fan BE, Ng J, Chan SSW, Christopher D, Tso ACY, Ling LM (2021). COVID-19 associated coagulopathy in critically ill patients: A hypercoagulable state demonstrated by parameters of haemostasis and clot waveform analysis. J Thromb Thrombolysis.

[12] Pavoni V, Gianesello L, Pazzi M, Stera C, Meconi T, Frigieri FC (2020). Evaluation of coagulation function by rotation thromboelastometry in critically ill patients with severe COVID-19 pneumonia. J Thromb Thrombolysis.

[13] Marone EM, Bonalumi G, Curci R, Arzini A, Chierico S, Marazzi G (2020). Characteristics of Venous Thromboembolism in COVID-19 Patients: A Multicenter Experience from Northern Italy. Ann Vasc Surg.

[14] Kaur S, Bansal R, Kollimuttathuillam S, Gowda AM, Singh B, Mehta D (2021). The looming storm: Blood and cytokines in COVID-19. Blood Rev.

[15] Ackermann M, Verleden SE, Kuehnel M, Haverich A, Welte T, Laenger F (2020). Pulmonary Vascular Endothelialitis, Thrombosis, and Angiogenesis in Covid-19. N Engl J Med.

[16] Manolis AS, Manolis TA, Manolis AA, Papatheou D, Melita H (2021). COVID-19 Infection: Viral Macro- and Micro-Vascular Coagulopathy and Thromboembolism/Prophylactic and Therapeutic Management. J Cardiovasc Pharmacol Ther.

[17] Vincent J-L, Levi M, Hunt BJ (2021). Prevention and management of thrombosis in hospitalised patients with COVID-19 pneumonia. Lancet Respir Med.

[18] Miesbach W, Makris M (2020). COVID-19: Coagulopathy, Risk of Thrombosis, and the Rationale for Anticoagulation. Clin Appl Thromb Hemost.

[19] Gomez K, Laffan M, Bradbury C. Debate: Should the dose or duration of anticoagulants for the prevention of venous thrombosis be increased in patients with COVID‐19 while we are awaiting the results of clinical trials? Br J Haematol. 2020. 10.1111/bjh.17241. 10.1111/bjh.17241 PMC7753713 33236402

[20] Al-Samkari H, Karp Leaf RS, Dzik WH, Carlson JCT, Fogerty AE, Waheed A (2020). COVID-19 and coagulation: bleeding and thrombotic manifestations of SARS-CoV-2 infection. Blood.

[21] EpiCentro. Sorveglianza integrata COVID-19: i principali dati nazionali. https://www.epicentro.iss.it/coronavirus/sars-cov-2-sorveglianza-dati. Accessed 9 Jan 2022.

[22] World Health Organization. Clinical management of COVID-19: interim guidance, 27 May 2020. World Health Organization; 2020. https://apps.who.int/iris/handle/10665/332196.

[23] Schünemann HJ, Cushman M, Burnett AE, Kahn SR, Beyer-Westendorf J, Spencer FA (2018). American Society of Hematology 2018 guidelines for management of venous thromboembolism: prophylaxis for hospitalized and nonhospitalized medical patients. Blood Adv.

[24] Cattaneo M, Bertinato EM, Birocchi S, Brizio C, Malavolta D, Manzoni M (2020). Pulmonary Embolism or Pulmonary Thrombosis in COVID-19? Is the Recommendation to Use High-Dose Heparin for Thromboprophylaxis Justified?. Thromb Haemost.

[25] Susen S, Tacquard CA, Godon A, Mansour A, Garrigue D, Nguyen P (2020). Prevention of thrombotic risk in hospitalized patients with COVID-19 and hemostasis monitoring. Crit Care Lond Engl.

[26] Warkentin TE, Greinacher A (2004). Heparin-induced thrombocytopenia: recognition, treatment, and prevention: the Seventh ACCP Conference on Antithrombotic and Thrombolytic Therapy. Chest.

[27] World Health Organization. Technical specifications for selection of essential in vitro diagnostics for SARS-COV-2, 14 June 2021. World Health Organization; 2021. https://apps.who.int/iris/handle/10665/341753.

[28] Binder DA (1992). Fitting Cox’s proportional hazards models from survey data. Biometrika.

[29] Austin PC (2011). An Introduction to Propensity Score Methods for Reducing the Effects of Confounding in Observational Studies. Multivar Behav Res.

[30] VanderWeele TJ (2019). Principles of confounder selection. Eur J Epidemiol.

[31] R Core Team (2021). R: A Language and Environment for Statistical Computing.

[32] Therneau TM (2021). A Package for Survival Analysis in R.

[33] Greifer N (2021). WeightIt: Weighting for Covariate Balance in Observational Studies.

[34] Iba T, Levy JH, Warkentin TE, Thachil J, van der Poll T, Levi M (2019). Diagnosis and management of sepsis-induced coagulopathy and disseminated intravascular coagulation. J Thromb Haemost.

[35] Flumignan RL, Civile VT, de Tinôco JDS, Pascoal PI, Areias LL, Matar CF (2022). Anticoagulants for people hospitalised with COVID-19. Cochrane Database Syst Rev.

[36] Minnai F, De Bellis G, Dragani TA, Colombo F (2022). COVID-19 mortality in Italy varies by patient age, sex and pandemic wave. Sci Rep.

[37] Meng B, Kemp SA, Papa G, Datir R, Ferreira IATM, Marelli S (2021). Recurrent emergence of SARS-CoV-2 spike deletion H69/V70 and its role in the Alpha variant B.1.1.7. Cell Rep..

[38] Stefanelli P, Lo Presti A, Ambrosio L, Di Martino A, Morabito S, Vaccari G (2021). Prevalenza e distribuzione delle varianti del virus SARS-CoV-2 di interesse per la sanità pubblica in Italia. Rapporto n. 2 dell’11 giugno 2021.

[39] Moores LK, Tritschler T, Brosnahan S, Carrier M, Collen JF, Doerschug K (2022). Thromboprophylaxis in Patients with COVID-19. A Brief Update to the CHEST Guideline and Expert Panel Report. Chest.

[40] Goligher EC, Bradbury CA, McVerry BJ, REMAP-CAP Investigators, ACTIV-4a Investigators, ATTACC Investigators (2021). Therapeutic Anticoagulation with Heparin in Critically Ill Patients with Covid-19. N Engl J Med.

[41] Lawler PR, Goligher EC, Berger JS, ATTACC Investigators, ACTIV-4a Investigators, REMAP-CAP Investigators (2021). Therapeutic Anticoagulation with Heparin in Noncritically Ill Patients with Covid-19. N Engl J Med.

[42] Pavoni V, Gianesello L, Pazzi M, Stera C, Meconi T, Frigieri FC (2020). Venous thromboembolism and bleeding in critically ill COVID-19 patients treated with higher than standard low molecular weight heparin doses and aspirin: A call to action. Thromb Res.

[43] Novelli C, Borotto E, Beverina I, Punzi V, Radrizzani D, Brando B (2021). Heparin dosage, level, and resistance in SARS-CoV2 infected patients in intensive care unit. Int J Lab Hematol.

